# Urine colour change as an indicator of change in daily water intake: a quantitative analysis

**DOI:** 10.1007/s00394-015-1010-2

**Published:** 2015-08-19

**Authors:** Erica T. Perrier, Evan C. Johnson, Amy L. McKenzie, Lindsay A. Ellis, Lawrence E. Armstrong

**Affiliations:** Danone Research, RD 128, 91767 Palaiseau Cedex, France; Human Performance Laboratory, University of Connecticut, Unit 1110, Storrs, CT 06269-1110 USA

**Keywords:** Hydration biomarkers, Fluid intake, Urine colour, Specific gravity

## Abstract

**Purpose:**

Urine colour (*U*_Col_) is simple to measure, differs between low-volume and high-volume drinkers, and is responsive to changes in daily total fluid intake (TFI). However, to date, no study has quantified the relationship between a change in TFI and the resultant change in *U*_Col_. This analysis aimed to determine the change in TFI needed to adjust 24-h *U*_Col_ by 2 shades on an 8-colour scale, and to evaluate whether starting *U*_Col_ altered the relationship between the change in TFI and change in *U*_Col_.

**Methods:**

We performed a pooled analysis on data from 238 healthy American and European adults (50 % male; age, 28 (sd 6) years; BMI 22.9 (sd 2.6) kg/m^2^), and evaluated the change in TFI, urine volume (*U*_Vol_), and specific gravity (*U*_SG_) associated with a change in *U*_Col_ of 2 shades.

**Results:**

The mean [95 % CI] change in TFI and *U*_Vol_ associated with a decrease in *U*_Col_ by 2 shades (lighter) was 1110 [914;1306] and 1011 [851;1172] mL/day, respectively, while increasing *U*_Col_ by 2 shades (darker) required a reduction in TFI and *U*_Vol_ of −1114 [−885;−1343] and −977 [−787;−1166] mL/day. The change in *U*_Col_ was accompanied by changes in *U*_SG_ (lighter urine: −.008 [−.007;−.010]; darker urine: +.008 [.006;.009]). Starting *U*_Col_ did not significantly impact the TFI change required to modify *U*_Col_ by 2 shades.

**Conclusions:**

Our results suggest a quantifiable relationship between a change in daily TFI and the resultant change in *U*_Col_, providing individuals with a practical means for evaluating and adjusting hydration behaviours.

## Introduction

Water is fundamental to sustaining life and has been referred to as the most essential nutrient [1, 2]. The link between hydration and overall health has recently been highlighted for a variety of health-related outcomes. For instance, plasma hypertonicity and low water intake are associated with hyperglycaemia, a precursor to insulin resistance and eventually type II diabetes [3, 4]. Separately, adults in the highest percentiles of daily fluid intake [5] and urine volume [6] have lower risk for developing chronic kidney disease (CKD), and low urine volume is also linked to the recurrence of kidney stone disease [7, 8]. Recent studies of adults in normal daily living conditions with ad libitum access to water have shown that habitual low-volume drinkers excrete low volumes of concentrated urine that correspond with the highest categories of CKD risk [6, 9]. Moreover, adults with low daily water intake show increases in circulating vasopressin (AVP), suggesting an osmotically driven release of AVP to increase water reabsorption in the kidney [9, 10]. Consuming a larger volume of water, enabling production of a greater volume of dilute urine is beneficial for the kidneys, urinary tract, and potentially other physiological systems [6, 8, 11, 12]. In this context, measures of urine concentration, including osmolality, specific gravity, and colour, are becoming increasingly relevant as biomarkers of not only acute hydration, but also of sufficient fluid intake for long-term health.

Among various urinary biomarkers of hydration, urine colour (*U*_Col_) is unique in its simplicity, cost, portability, and minimal required technical expertise [13]. While originally validated by Armstrong and colleagues as a way to monitor acute dehydration in athletic populations [14, 15], the utility of *U*_Col_ has since been expanded to include monitoring hypohydration in average adults during activities of normal daily living. Urine colour has been shown to differ between habitual low-volume and high-volume drinkers [9, 16, 17], and is responsive to changes in daily total fluid intake [18]. However, to date, no study has quantified the relationship between a change in daily total fluid intake (TFI) and the resultant change in *U*_Col_ in young, healthy adults. Understanding this relationship would provide individuals with a practical means for evaluating, modifying, and monitoring their hydration behaviours, by identifying the volume increase in daily TFI that is necessary to adjust *U*_Col_ from a darker to a lighter shade.

This analysis had two specific aims. The first was to determine the change in daily fluid intake volume needed to adjust *U*_Col_ by 2 shades on Armstrong et al.’s [14] 8-colour scale. We also speculated that individuals’ baseline *U*_Col_ might impact the volume of fluid intake change necessary to elicit a two-shade change in *U*_Col_ (i.e. an individual shifting from *U*_Col_ of 6–4 might require a smaller TFI increase than an individual shifting from *U*_Col_ of 4–2). This speculation was inspired by chemistry dilutions, where the relationship between change in volume and change in concentration is not linear. Thus, our second aim was to evaluate whether starting *U*_Col_ (dark vs. moderate) altered the amount of fluid required to elicit a change in *U*_Col_.

## Methods

We performed a pooled analysis of four independently conducted studies. All four studies were conducted according to the Declaration of Helsinki and were approved by the appropriate ethics committees, and all subjects provided informed consent. Together, the four studies reflected a variety of study settings, interventions (increasing daily TFI in low drinkers, decreasing daily TFI in high drinkers, or maintaining daily TFI habits), and drinking habits representative of typical North American and Western European consumption habits (Table 1). The pooled database included 238 participants, 50 % male, with a broad representation of daily TFI (5th–95th percentile: 0.37–3.22 L/d) and corresponding 24-h urine samples.

**Table 1:** Study description: setting, interventions, duration, and participants

	Study A^a^	Study B^b^	Study C^c^	Study D^d^	Total
Setting	Ambulatory	Inpatient	Ambulatory	Ambulatory	
Intervention(s)	No intervention; maintain normal drinking habits	Increase daily water intake in low-volume drinkers	Increase daily water intake in low-volume drinkers	Increase daily water intake in low-volume drinkers	
		Decrease daily water intake in high-volume drinkers	Maintain normal drinking habits in low-volume drinkers	Decrease daily water intake in high-volume drinkers	
Duration	4 consecutive days	5 consecutive days	7 weeks	7 consecutive days	
Participants (*n*)	96	52	62	28	238
Men/women (%)	49/51	21/79	100/0	0/100	50/50
Age (years)	32 ± 4	25 ± 3	29 ± 7	20 ± 2	28 ± 6
BMI (kg/m^2^)	23.3 ± 2.8	22.4 ± 1.7	22.9 ± 2.7	22.5 ± 3.0	22.9 ± 2.6
Day 1 TFI (L/d)	1744 ± 1095	1635 ± 748	597 ± 169	1939 ± 1007	1443 ± 991
TFI (5th–95th pct; L/d)					373–3217

^a^ *Study A* [9]. During four consecutive weekdays, sedentary French adults with a broad range of daily TFI behaviours continued with their normal daily activities, maintained their normal eating and drinking habits, and completed a daily online food and fluid intake diary. Three consecutive 24-h urine samples were collected during this period and were returned to the investigating site each morning

^b^ *Study B* [18]. During five consecutive days, participants with either habitual low-volume drinking (LD; TFI 0.71 ± 0.28 L/d) or high-volume drinking (HD; TFI 2.66 ± 0.65 L/d) habits completed an inpatient, crossover trial. On the first 2 days, participants were prescribed a daily water intake volume similar to their normal habits (LD: 1.0 L/d; HD: 2.5 L/d). On the following 3 days, intake volumes were reversed between groups (LD: increased intake to 2.5 L/d; HD: intake restricted to 1.0 L/d). Five consecutive 24-h urine samples were collected during the inpatient trial

^c^ *Study C* [26]. Habitual low-volume drinkers were assigned either to a control group or to a 7-week water intake intervention designed to increase TFI (+1.5 L/d plain water on top of their normal daily fluid intake). During three consecutive days at baseline and again at the end of the intervention, participants completed an online food and fluid intake diary and collected two 24-h urine samples (one at baseline and one at the end of intervention)

^d^ *Study D* [10]. Habitual low-volume (LD; total water from food and fluids 1.62 ± 0.48 L/d) and high-volume (HD; total water from foods and fluids 3.34 ± 0.56 L/d) drinkers maintained their normal daily intake habits over two consecutive days, followed by a 4-day intervention in which LD increased their intake to 3.0 L/d of plain water, while HD were restricted to 1.25 L/d of plain water. After the 4-day intervention, participants returned to ad libitum fluid intake for 1 day. Participants completed a daily food and fluid intake diary and collected seven consecutive 24-h urine samples

### Participant lifestyle habits and relevant inclusion criteria

Lifestyle habits and relevant inclusion or non-inclusion criteria, which may impact fluid balance and homeostasis, were reviewed prior to analysis. In all studies, participants were sedentary or participated in only light-to-moderate physical activity. In the case in which some physical activity was permitted [10], exercise logs were kept to ensure that physical activity was within the prescribed limits (actual physical activity reported: 1 ± 1 sessions during the intervention with an average duration of 25 ± 18 min/session). Caffeine intake of ≤500 mg/day was permitted in 3 of the 4 studies. This level of daily caffeine intake has been shown, in free-living young adults, to cause no dehydration or measurable differences in hydration indices [19]. In all studies, exclusionary criteria included metabolic or gastrointestinal disease (acute or chronic), renal, hepatic, or cardiac failure, and any drug or concomitant medication that may interfere with renal function or water balance.

### Total fluid intake

In all four studies, intake was recorded throughout each study day. Daily TFI was defined as the total volume of drinking water plus other beverages. In studies A and C, fluid intake data were collected via an online food and fluid intake diary, which participants filled out daily (NutriSaas-24WQ-waters; MXS, France) and which included questions specific to fluid intake both during and between meals. Study B was conducted using an inpatient setting, meaning that food and water consumption was controlled, monitored, and recorded by the study staff. In Study D, subjects filled out daily paper food and fluid diaries, which were double-checked for completeness via interviews with trained staff each morning and were analysed using commercial nutrition software (Nutritionist Pro, Axxya Systems, Redmond, WA).

### Twenty-four-hour urinary variables

In studies A, B, and C, 24-h urine volume (*U*_Vol_) was measured from urine mass (to the nearest gram) and corrected for density using specific gravity. In study D, urine volume was measured from urine mass alone.

In studies A, B, and C, 24-h urine specific gravity (*U*_SG_) was measured using a commercially available digital hand-held refractometer (ATAGO Pen Urine-SG, Atago Corp., Japan). In study D, *U*_SG_ was measured using a clinical light refractometer (ATAGO A300CL, Atago Corp., Japan).

All four studies evaluated 24-h *U*_Col_ using the 8-shade urine colour scale published by Armstrong et al. [20]. In all four studies, a transparent urine collection container was placed against a plain white background in a well-lit room. The colour of the sample was compared against the colour scale, and the number corresponding to the closest shade (1 = very pale; 8 = very dark) was recorded.

### Data recombination and analysis

Extractions of demographic, TFI, and urine variables were performed on each of the study databases; variable names were recoded, and the extractions were merged into a common database. For each participant, between-day change scores were calculated for TFI, *U*_Vol_, *U*_SG_, and *U*_Col_. In studies A, B, and D, where variables were collected over consecutive days, change scores reflect the changes in TFI and urinary parameters between consecutive 24-h periods. In study C, variables were collected at baseline and upon completion of the intervention, and thus change scores reflect the change between baseline and intervention periods. Because the database included studies where participants were asked to increase, decrease, or maintain their daily fluid intake, data were then grouped by the directionality of the *U*_Col_ change score (i.e. *U*_Col_ became lighter; *U*_Col_ became darker; or no change in *U*_Col_). These three data groups were then analysed separately. For each data group, the respective changes in TFI, *U*_Vol_, and *U*_SG_ that were associated with a change in *U*_Col_ were calculated.

To determine the change in daily fluid intake volume needed to adjust *U*_Col_ by 2 shades (Aim 1), two separate approaches were used to evaluate the change in TFI required to modify *U*_Col_ by 2 shades. In the first approach, all data points displaying the same change in *U*_Col_ were grouped, and the mean change in TFI, *U*_Vol_, and *U*_SG_ that was required to modify *U*_Col_ by ± 1, 2, 3, or 4 shades was calculated. In the second approach, change in *U*_Col_ was recoded as a binomial variable (0: change in *U*_Col_ < 2 shades; 1: change in *U*_Col_ ≥ 2 shades) and receiver operating characteristic (ROC) analysis was used to determine the ‘optimal’ change in TFI associated with a change of 2 or more *U*_Col_ shades.

To evaluate whether starting *U*_Col_ altered the relationship between the change in fluid intake and change in *U*_Col_ (Aim 2), it was also necessary to consider two cases (*U*_Col_ becoming lighter; *U*_Col_ becoming darker) separately. This separation was necessary because subjects with 24-h *U*_Col_ that was already relatively dark (shades 5 or 6 on the 8-colour scale) would be unlikely to exhibit even darker 24-h urine the following day, while subjects with very pale urine (1 or 2) would not be able to lighten their *U*_Col_ by 2 or more shades on the 8-point scale. Thus, for data points where *U*_Col_ became lighter, we compared change in TFI between urine starting at a moderate colour (shades 3 or 4), to urine starting at a darker colour (shades 5 or 6). Urine samples starting at very dark shades (7 or 8) could not be assessed since there were only four samples that fell in this colour range. For data points where *U*_Col_ became darker, we compared the change in TFI between urine starting at a very pale colour (shades 1 or 2), to urine starting at a moderate colour (shades 3 or 4).

## Results

### Aim 1. Change in daily fluid intake volume needed to adjust urine colour by 2 shades on an 8-colour urine colour scale

**Fig. 1:**
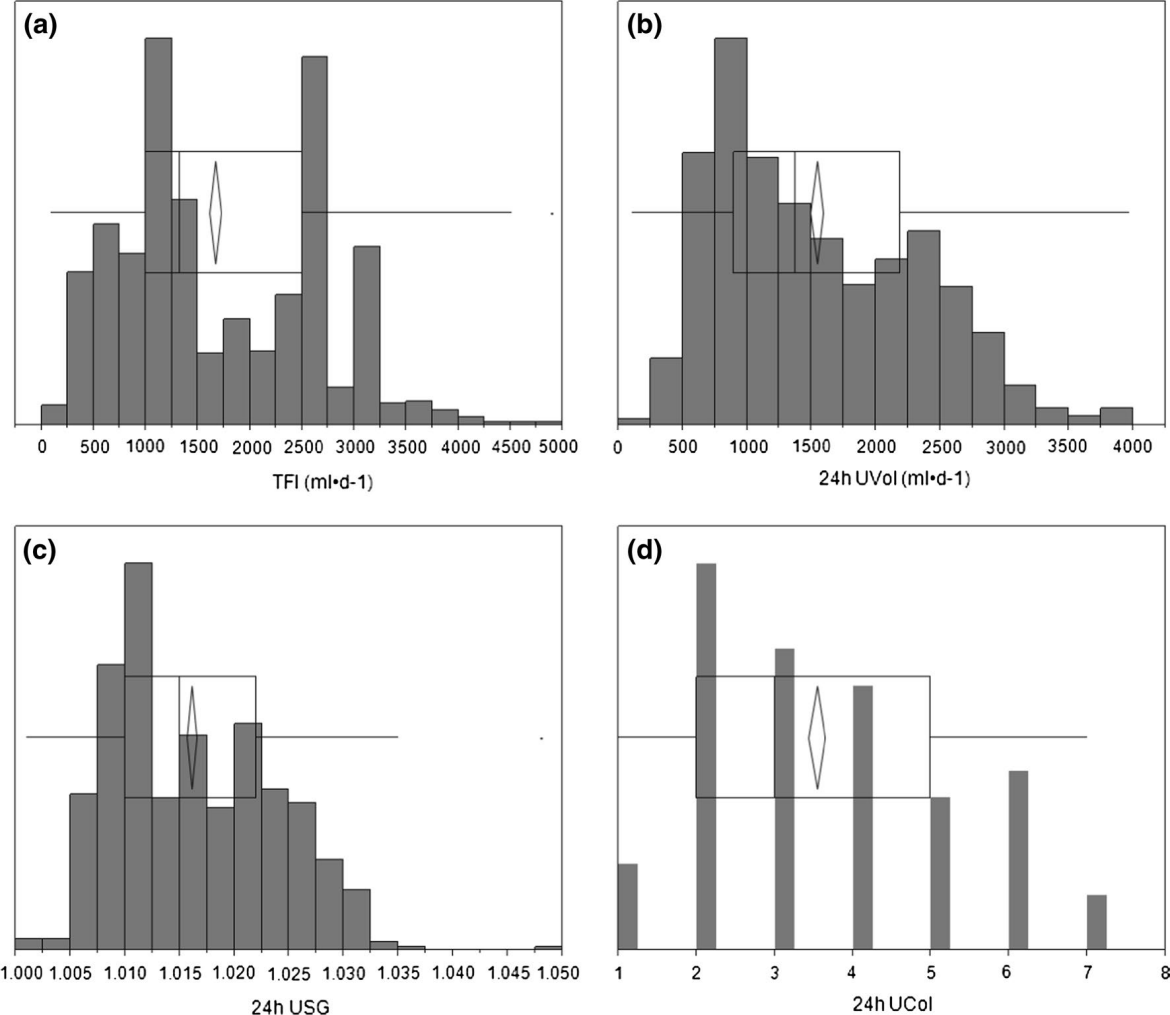
Distribution of **a** TFI, **b** 24-h *U*_Vol_, **c** 24-h *U*_SG_, **d** 24-h *U*_Col_

**Fig. 2:**
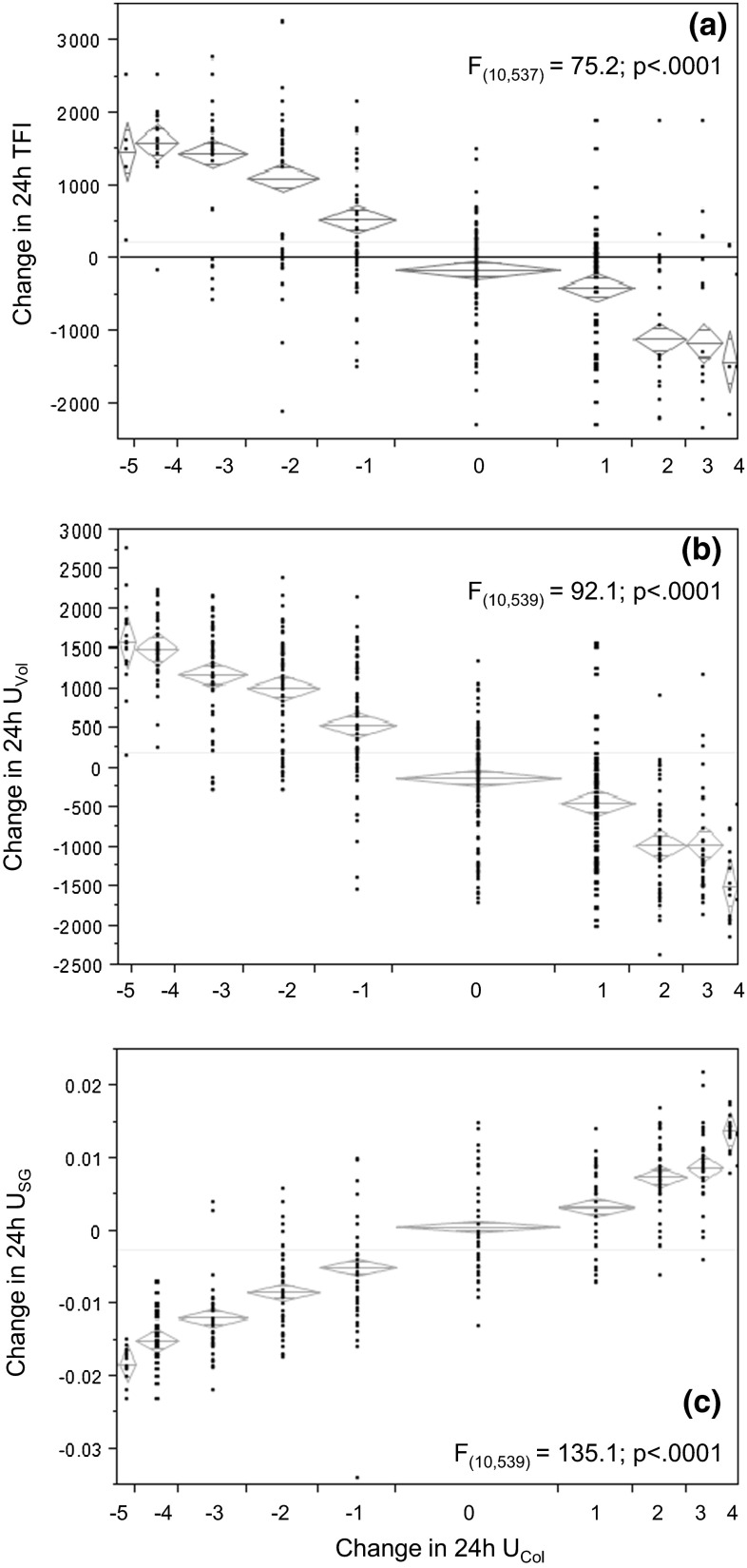
Change in daily **a** TFI, **b** 24-h *U*_Vol_, and **c** 24-h *U*_SG_, respectively, as a function of change in 24-h *U*_Col_

The distributions of TFI and urine variables are shown in Fig. 1. For the first approach, the mean change in TFI, *U*_Vol_, and *U*_SG_ required to modify *U*_Col_ by ±1, 2, 3, 4, and 5 shades was calculated after pooling all participant data. This database included 144 instances of no change in *U*_Col_, 245 instances in which *U*_Col_ decreased (change = −1,−2,−3,−4, or −5 shades, lighter), and 159 instances in which *U*_Col_ increased (change = +1, +2, +3, or +4 shades, darker). The mean [95 % CI] change in TFI required to decrease *U*_Col_ by precisely 2 shades (lighter colour) was 1110 [914;1306] mL/day, while increasing *U*_Col_ by precisely 2 shades (darker colour) required a decrease in TFI of 1114 [−885;−1343] mL/day. These changes in TFI were supported by changes in 24-h *U*_Vol_ (lighter urine: +1011 [851;1172] mL/day; darker urine:−977 [−787;−1166] mL/day) and *U*_SG_ (lighter urine:−.008 [−.007;−.010]; darker urine: +.008 [.006;.009]). Figure 2 illustrates the change in daily (a) TFI, (b) 24-h *U*_Vol_, and (c) *U*_SG_, respectively, as a function of change in U_Col_.

**Fig. 3:**
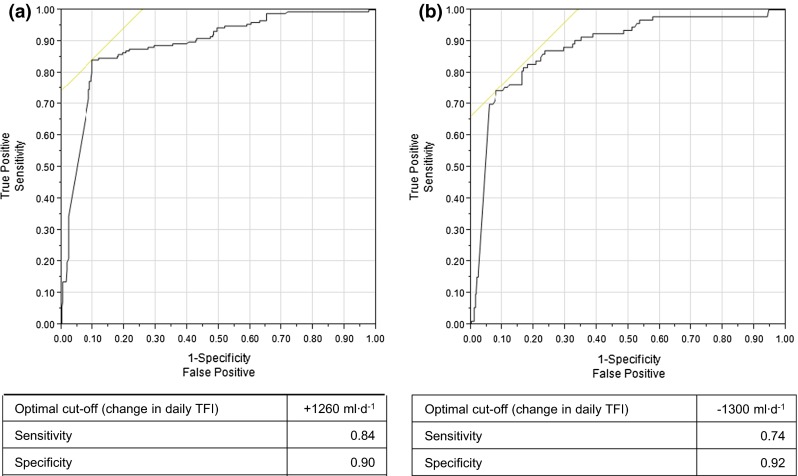
Receiver operating characteristic curves assessed the optimal change in TFI to **a** lighten 24-h *U*_Col_ by at least 2 shades and **b** darken 24-h *U*_Col_ by at least 2 shades. The area under the curve was (**a**) 0.89 and (**b**) 0.88, respectively

For the second approach to determining the TFI change that is required to change *U*_Col_ by 2 shades, logistic regression curves were generated for positive and negative changes in *U*_Col_. Two separate ROC analyses were performed with a positive outcome that was defined as a *U*_Col_ change of ≥2 shades (Fig. 3). The optimal cut-off in ΔTFI to generate a decrease in *U*_Col_ by at least 2 shades was by consumption of an additional 1260 mL/day (AUC = 0.89), while an increase in *U*_Col_ was optimally achieved by a reduction in fluid consumption of −1300 mL/day (AUC = 0.88).

### Aim 2. Evaluate whether starting *U*_Col_ influences the change in TFI that is required to change *U*_Col_

To address the second aim, only data points where 24-h *U*_Col_ changed by exactly 2 shades between days were included. The database included 59 instances when *U*_Col_ decreased by 2 shades (lighter colour) and 46 instances when *U*_Col_ increased by 2 shades (darker colour). To evaluate whether starting U_Col_ affected the TFI volume required to decrease (lighten) *U*_Col_ by 2 shades, we tested for differences in mean TFI change between urine starting at a moderate colour (shades 3 or 4), to urine starting at a darker colour (shades 5 or 6). There was no significant difference in TFI change [1255 (sd 997) vs. 1092 (sd 877) mL/day, *p* = 0.52] or *U*_Vol_ change [1164 (sd 632) vs. 948 (sd 714) mL/day, *p* = 0.24] between samples starting at a moderate versus dark shade.

In the second instance, where *U*_Col_ became darker, we compared the average decrease in TFI between very dilute urine samples (shades 1 or 2), versus urine starting at a moderate shade (shades 3 or 4). No significant differences were detected in the TFI change [−1310 (sd 815) vs.−892 (sd 795) mL/day, *p* = 0.09] or *U*_Vol_ change [−1101 (sd 680) vs.−838 (sd 677) mL/day, *p* = 0.20] needed to modify *U*_Col_ by 2 shades.

## Discussion

Daily water needs are highly individual and depend upon body composition and size, dietary solute intake, physical activity and fitness level, and other factors such as climate, environment, and disease. In order to monitor the adequacy of their hydration behaviours, individuals can track daily intake, but this type of tracking remains based upon achieving the general population requirement and is not dynamic (i.e. does not adjust for differences in climate, diet, and physical activity). A second option for individual hydration monitoring is to track urine output. Urine output, and more specifically urine concentration, is the end result reflecting the renal regulation required to maintain water balance in response to varying levels of water intake and loss. Thus, measures of urine concentration have greater utility for the individual assessment of daily water intake, as differences in daily total water intake, sweat loss, dietary solute load, and climate are all reflected in the volume and concentration of 24-h urine.

The urine colour scale correlates with other, more sophisticated measures of urine concentration in situations of acute dehydration [14, 15] as well as in sedentary adults in normal daily living conditions [16, 17, 21]. Significant changes in urine colour occur within 24 h of modifying fluid intake volume [18], suggesting that individuals can use urine colour monitoring as a simple way of evaluating the adequacy of their fluid intake, and consequently making adjustments that result in visibly lighter urine.

The results of this study suggest that a change in urine colour by 2 shades can be achieved with a quantifiable change (1100–1300 mL/day) in daily water intake. Moreover, the change in daily water intake and the change in urine colour shades were supported by corresponding changes in urine volume (1.0 L/d) and specific gravity (0.008 units), respectively. To demonstrate this, we chose two analytical approaches. The first approach was to determine the scaling of urine colour change, (i.e. What is the volume of additional water intake required to change urine colour by one, two, or more shades?) The second approach (ROC) provided a more clinical perspective, (i.e. How much additional water intake is needed to lighten a person’s urine colour by at least 2 shades over a 24-h period?). From a practical perspective, these findings may serve to provide fluid intake guidance for individuals producing relatively low volumes of concentrated urine. For instance, maintaining a high urine volume [22] with target urine specific gravity of <1.010 [23] is recommended as a cornerstone in the prevention of recurrent kidney stones. Moreover, establishing an individual daily intake requirement associated with a target urine concentration was the approach used by the European Food Safety Authority (EFSA), who argued that the water intake recommendation should target a urine osmolarity of approximately 500 mOsm/L in order to provide a safe margin of free water reserve [24]. From this sensitivity analysis, we can expect that 84 % of individuals with darker urine colour who increase TFI by at least 1260 mL over the course of the day will see a two-shade decrease in 24-h *U*_Col_. Given the reduced risk of kidney complications associated with higher volumes of dilute urine [6–8], it would seem advantageous for individuals with habitual darker urine colour to strive for this two-shade reduction in urine colour via increased daily fluid intake.

This study has several strengths. First, the changes in total fluid intake and urine colour were supported by corresponding and coherent changes in urine volume and specific gravity. Moreover, the changes were bidirectionally symmetrical, were observed in both men and women, and were applicable across a wide range of TFI representing the consumption habits of a large proportion of the American and European population. The mean change in total fluid intake to change urine colour by 2 shades was also supported by receiver operating characteristic curves with high AUC, sensitivity, and specificity. The most substantial limitations of this retrospective analysis were that (1) the studies that were pooled employed interventions with fairly large changes in daily water intake, and (2) there was some heterogeneity in the pooled studies. We consider that this analysis was a first step, but that the exercise must be repeated with a prospective design which would allow for a more comprehensive evaluation of the effect of small changes (<1 L per day) in daily water intake on urine concentration and volume, as well as evaluate the time course required to achieve a noticeable change in urine colour. Moreover, it is important to note that urine-concentrating capacity diminishes naturally with age, and therefore urinary biomarkers of hydration have limited value in the elderly. A recent study by Fortes et al. [25] reported that in a sample of patients with a mean age of 79 years, neither urine colour nor specific gravity was able to accurately distinguish euhydrated and dehydrated patients. Indeed, the restriction of range resultant from age-related reduction in urine-concentrating capacity is evident in this sample, where dehydrated, elderly patients only achieved a mean urine specific gravity of 1.017 and a colour of 4.

In conclusion, our results suggest a quantifiable relationship between a change in daily water intake and the resultant change in urine colour. Understanding this relationship would provide individuals with a practical means for evaluating and modifying their hydration behaviours, by identifying the volume increase in daily water intake that is necessary to adjust urine colour from a darker to a lighter shade.
